# Effect of housing construction material on childhood acute respiratory infection: a hospital based case control study in Bangladesh

**DOI:** 10.1038/s41598-024-57820-6

**Published:** 2024-04-08

**Authors:** Jhantu Bakchi, Rosul Ahmed Rasel, Khandokar Farmina Shammi, Sumaiya Ferdous, Shamima Sultana, Mst. Rokshana Rabeya

**Affiliations:** Department of Public Health Nutrition, School of Science, Primeasia University, Dhaka-1213, Bangladesh

**Keywords:** Environmental impact, Medical research, Risk factors

## Abstract

Despite several studies conducted to investigate housing factors, the effects of housing construction materials on childhood ARI symptoms in Bangladesh remain unclear. Hence, the study aimed to measure such a correlation among children under the age of five. A hospital-based case–control study was conducted, involving 221 cases and 221 controls from January to April 2023. Bivariate and multivariate binary logistic regression was performed to measure the degree of correlation between housing construction materials and childhood ARI symptoms. Households composed of natural floor materials had 2.7 times (95% confidence interval 1.27–5.57) and households composed of natural roof materials had 1.8 times (95% confidence interval 1.01–3.11) higher adjusted odds of having under-five children with ARI symptoms than household composed of the finished floor and finished roof materials respectively. Households with natural wall type were found protective against ARI symptoms with adjusted indoor air pollution determinants. The study indicates that poor housing construction materials are associated with an increased risk of developing ARI symptoms among under-five children in Bangladesh. National policy regarding replacing poor housing materials with concrete, increasing livelihood opportunities, and behavioral strategies programs encouraging to choice of quality housing construction materials could eliminate a fraction of the ARI burden.

## Introduction

In Bangladesh between 2015 and 2018, the under-five mortality rate decreased from 46 to 45 deaths per 1000 live births which allowed the country to meet Millennium Development Goal (MDG) 4. In 2016, 1.87 million new cases of pneumonia were recorded worldwide and Bangladesh was among the five countries that account for the majority of pediatric pneumonia cases^1,2^. The Bangladesh Sample Vital Statistics 2018 reported pneumonia as the predominant reason for under-five fatalities, accounting for over 34% of all deaths^3^. The shreds of evidence show acute respiratory infection, particularly pneumonia, is still a major cause of illness among under-five children. Therefore, Bangladesh must take care of the potential risk factors of ARIs to achieve the objective of the integrated Global Action Plan for the Prevention and Control of Pneumonia and Diarrhea (GAPPD) by 2025, which set the target of lowering the pneumonia mortality rate to fewer than three per 1000 live-births^4^ and to meet Sustainable Development Goal (SDG) 3, that aims to put an end to the preventable deaths of infants and under-five children worldwide^5^. ARI morbidity and mortality are correlated to several risk factors., including malnutrition^6^, Gestational age^7^, inadequate breastfeeding^8^, over-crowding^7^, indoor and outdoor pollution^9^, seasonality^10^, poor housing conditions^11^, and lack of quality medical facilities^12^. In Bangladesh, children with ARIs are also exposed to several risk factors of these. Many previous investigators have examined the risk factors of ARIs like indoor and outdoor air pollution that explains the exposures that are harmful to health from smoking^7^, inhouse cooking activities, and culinary activity seeping into the home's surrounding areas^9,13^. The US Environmental Protection Agency (EPA) states that the rate at which air circulates between inside and outside is a crucial component in determining the amounts of indoor air pollutants in a house. The building's construction, design, materials, and operating characteristics have an impact on the air exchange rate. Eventually, natural ventilation, mechanical ventilation, and infiltration—air entering buildings through gaps and fractures in the windows, floors, partition walls, roof, and doors—all directly affect the air exchange rate^14^. Regarding the building materials used in homes, studies from Pakistan^11^, India^15^ and Nigeria^16^ have shown that children who live in houses composed of poor construction materials like hard-packed mud and thatch have higher risk of having health outcomes in contrast to those who reside in houses with concrete foundations, tile roofs, and walls made of fired mud brick. Therefore, housing construction materials may contribute to high rates of childhood ARI in Bangladesh.

Even though Bangladesh has a higher rate of urbanization^17^, many of its citizens live in substandard housing because of the country's greatest population growth. According to The Multiple Indicator Cluster Survey (MICS) 2019, 60.8% of households are composed of natural floor materials (Earth/Sand, and Dung), 12.3% of households are composed of natural wall materials (cane/palm/trunks, dirt, bamboo with polyethylene) and 0.7% of natural roof materials (thatch/palm leaf/ nipa palm, sod)^18^. More practically, about 84.8% of total households use “Tin” as roofing materials^19^.“Tin” as roofing materials may act as a hidden risk factor for increased childhood ARI rate which has remained unspoken. Moreover, the impact of household construction materials on childhood ARI didn’t get proper attention to be considered as a potential risk factor for increased ARI rate in Bangladesh. In the context of quality housing construction, one of the studies analyzed the Bangladesh Demographic and Health Survey-2017–2018 data concluded that improved housing infrastructure may lead to the use of clean and safer fuel to reduce ARI burden and another study explored only floor material as a risk factor for childhood ARI while the role of wall and roof material remain neglected^13,20^. No study using primary or secondary data has been found to investigate the effect of housing infrastructure in terms of household floor, wall, and roof materials and their joint effects on childhood ARIs keeping it in mind as a primary objective. Besides, to the authors’ knowledge, no such study has been conducted in light of the case–control study design. Hence, the primary objective of this study is to shed light on the contribution of housing infrastructure considering it as a suspected risk factor for increased childhood ARI rates and, reduce the literature gap.

Considering the housing structure indicators derived from the MICS questionnaire to provide clear evidence, the study aimed to measure the correlation between household construction materials and childhood ARI. Findings from the study will provide evidence to policymakers for undertaking comprehensive programs to reduce the ARI burden through better quality housing and housing finance.

## Methods

### Study site

A hospital-based case–control study was conducted from January to April 2023, at Dr M R Khan Shishu Hospital, Dhaka under Shishu Sasthya Foundation Bangladesh. The hospital provides services through its 175 bedded in-patient departments and other departments including the Paediatric Medicine Department, Paediatric Surgery, Special Care and Neonatal Unit (SCANU), and Diagnostic Division. Around 650–700, paediatric patients receive treatment daily in the outpatient department and emergency unit. Due to the affordable medical costs, patients from all over the country visit the hospital. However, this study was conducted in the pediatric medicine and outpatient departments of the hospital.

### Selection of case and controls

Among under-five children who had recently been admitted, had relapsed, or had been readmitted to the Dr M R Khan Shishu Hospital, Dhaka with ARI were considered as cases. ARI among children was assessed by following the IMCI protocol by hospital physicians^21^. The ability of the children to drink enough, cough or breathing difficulties, chest indrawing, stridor in normally quiet children, abnormal sleepiness or difficulty waking, convulsions, and fever or low body temperature were taken into account to identify potential ARI cases. The controls were non-ARI attendees from the outpatient department who hadn't had ARI in the last 30 days. Healthy and normal children who happened to be visiting with their parents in outpatient departments were also considered as controls. Children with chronic disorders (e.g., birth defects, tuberculosis, etc.) were not taken either as a case or control.

### Sample size estimation

The sample size was calculated assuming a 5% significance level, 80% power, a case–control ratio of 1:1, and a least extreme odd ratio (OR) of 2.0. The proportion of controls with household quality-natural roof type (73.52%) was estimated from a study conducted in Pakistan^11^. These assumptions required a minimum sample size of 223 cases and 223 controls. The sample size was calculated based on the formulas of Fleiss method with the continuity-correction factor^22^.

First, the sample size was calculated without the continuity-correction factor by Fleiss method. The formula is as below-

$${\rm{N}}_{{\rm{Fleiss - cases}}} = \, \left\{ {{\rm{Z}}_{{{{\upalpha /2}}}} \surd \left( {\left( {{\rm{r}} + 1} \right){\rm{ pq}}} \right) \, + {\rm{Z}}_{{{\rm{1 - \beta }}}} \surd \left( {\rm{rp1q1 + p2q2}} \right)} \right\}^{2} /{\rm{r}}\left( {\rm{p1 - p2}} \right)^{2}$$

where,

N_Fleiss-cases_ = Number of cases.

Z_α/2_ = Standard normal deviate for a two-tailed test based on alpha level.

Z_1- β_ = Power.

r = Ratio of controls to cases.

OR = Extreme odd ratio

p2 = Proportion of controls with exposure

q2 = 1–p2.

p1 = Proportion of cases with exposure.

p1 = (p2 OR)/{(1 + p2(OR–1)} (The proportion of cases exposed is calculated since the OR was provided).

q1 = 1–p1.

p = (p1 + rp2)/(r + 1) and.

q = 1-p.

Therefore, the number of cases without continuity-correction were- = 205.17≈205.

For the Fleiss method with the continuity-correction factor, the sample size from the uncorrected sample size formula was placed into the following formula

$${\rm{N}}_{{{\rm{Fcc}}}} = \left( {{\rm{N}}_{{\rm{Fleiss - cases}}} /4} \right) \, \left[ {1 + \surd \left\{ {1 + (\left( {2\left( {{\rm{r}} + 1} \right)/ \, \left( {{\rm{N}}_{{\rm{Fleiss - cases}}} {\rm{r}}*{\rm{r}}*|{\rm{p}}2 - {\rm{p}}1|} \right)} \right)} \right\}} \right]^{2}$$

where,

N_Fcc_ = Number of cases with the continuity-correction factor.

Fcc = Fleiss continuity-correction (approximate discrete distribution from continuous distribution).

The final case number with the continuity-correction were- = 222.42≈223.

However, 221 cases and 221 controls were included in the study as 2 cases and 2 controls were found to have incomplete responses prior to data cleaning and sorting.

### Data collection technique and quality control

A semi-structured questionnaire was used to collect data through face-to-face interviews under the guidance of hospital physicians. Three experienced and trained data collectors who have completed a diploma in Medical Assistant (MATS) from Bangladesh were involved in data collection to increase the quality and acceptability of data. A one-day training session was given to the data collectors on the objective and protocol of face-to-face interviews. A pilot study was conducted on twenty respondents in the same hospital and necessary changes were corrected in the questionnaire where applicable. The questionnaire was described in both English and Bengali languages to better understand the factual answers of the participants.

### Housing infrastructure

In this study, the housing infrastructure defines the nature and type of construction materials used to build the floor, wall, and roof of the house. The most common measure used in assessing the housing infrastructure in terms of constructing materials was described in the Multiple Cluster Indicator Survey (MICS). Multiple Indicator Cluster Surveys (MICS) are household surveys that are carried out by countries as part of the United Nations Children's Fund (UNICEF) effort to produce globally comparable, statistically sound data on the situation of women and children^23^. Table 1 shows the survey questionnaire of housing characteristics classification in terms of construction materials as natural, rudimentary, and finished used in the Bangladesh MICS-2019 report^18^.

**Table 1:** Categorization of housing construction materials according to MICS-2019.

	Flooring type	Wall type	Roof type
Natural	Earth/Sand, Dung	No wall, Cane / Palm / Trunks, Dirt, Bamboo with Polyethene	No roof, Thatch / Palm Leaf/ Nipa Palm, Sod
Rudimentary	Wood Planks, Palm / Bamboo/Betel Nut	Bamboo with Mud, Stone with Mud, Uncovered Adobe, Plywood, Cardboard, Reused Wood, Tin	Rustic Mat, Palm / Bamboo
Finished	Parquet Or Polished Wood, Vinyl or Asphalt Strips, Ceramic Tiles, Cement, Carpet	Cement, Stone with Lime / Cement, Bricks, Cement Blocks, Covered Adobe, Wood Planks / Shingles	Metal/Tin, Wood, Calamine / Cement Fibre, Ceramic Tiles, Cement, Roofing Shingles

Table 2 shows the modified categorization of housing materials used in this study as natural and finished. Rudimentary and natural were considered as one category. This categorization supports the study conducted in Pakistan^11^. The MICS-2019 survey report of Bangladesh shows that only 0.7% of the households had natural roofing and 0.1% of households had rudimentary roofing^18^. On the other hand, the Bangladesh Maternal Mortality and Health Care Survey-2016 (BMMS) report shows that about 84.8% of households used “Tin” as a roofing material^19^. To better understand the effect of roofing materials on ARI “Tin” was categorized purposively as a natural product against the finished product in this study. Thus, the floor, wall, and roof were categorized into: finished floor as 0 and natural floor as 1, finished wall as 0 and natural wall as 1, and finished roof as 0 and natural roof as 1.

**Table 2:** Modified categorization of natural and finished construction materials.

Category	Floor Type	Wall type	Roof type
Natural	Earth/Sand, and Dung	No walls, cane/palm/trunks, dirt, bamboo with mud, stone with mud, uncovered adobe, plywood, cardboard and reused wood	No roof, tin, thatch/palm leaf, sod, rustic mat, palm/bamboo, and wood planks
Finished	Parquet or polished wood, vinyl or asphalt strips, ceramic tiles/marble/chips, cement, carpet, and bricks floor	Cement, Stone with lime/cement, bricks, cement blocks, and covered adobe	Metal /t-iron/girders, wood/wooden beams, calamine/cement fiber, ceramic tiles and cement

### Composite indicators of housing infrastructure

Using composite variables is a common practice for organizing multiple highly correlated variables into more digestible or meaningful information^24^. Based on the housing infrastructure categories three composite variables were created and coded as follows (Table 3).

**Table 3:** Composite indicator of housing infrastructure.

Floor and wall	Wall and roof	Floor and roof
Finished floor + finished wall-0	Finished wall + finished roof-0	Finished floor + finished roof-0
Natural floor + natural wall-1	Natural wall + natural roof-1	Natural floor + natural roof-1
Finished floor + natural wall-2	Finished wall + natural roof-2	Natural floor + Finished roof-2
Natural floor + finished wall-3	Natural wall + finished roof-3	Finished floor + natural roof-3

### Independent variables

Data were collected on four sets of predictors: socio-demographic, housing characteristics, maternal and child factors, and indoor air pollution. Socio-demographic variables included the age and sex of the child, parental education and profession, and household monthly income. Parental education was categorized as 0(No formal education), 1 (Primary level education), 2(Secondary level education), 3 (Higher secondary level education) and 4 (Bachelor or higher education). Household monthly income was categorized as ≤ 10,000 as 0, 10,001 to 25,000 as 1, and ≥ 25,000 as 2.

Housing characteristics included residence (urban vs rural), number of bedrooms, source of drinking water, and types of toilet facilities. The source of drinking water was categorized as unimproved (Pond water, Borehole) as 0 vs. Improved (Pipped Water in house, Pipped into dwelling, and Reserve water). Toilet facilities were categorized as Improved (Ventilated improved pit latrine, Pit latrine with slab) as 0 vs. Unimproved (Bucket toilet, hanging toilet/slab Latrine without a water seal, hanging toilet/Hanging latrine, and No facility/Bush/Field) as 1. In-house crowding was determined by counting the number of people per bedroom (PPB)^7^. A PPB greater than 3 is considered an overcrowded house.

Maternal and child factors included gestational age (preterm- delivery of child before 37 weeks of gestation or full-term- delivery of the child after 37 weeks of gestation), mode of delivery (caesarean section or normal), initiation of breastfeeding (within one hour/ after one or two days), nutritional status and vaccination (completeness for age or no/incomplete). MUAC tape was used to assess the nutritional status and categorized as normal (MUAC ≥ 12.5 cm), moderately malnourished (MUAC- 11.5 to 12.4 cm), and severely malnourished (MUAC ≤ 11.5 cm).

Indoor air pollution was assessed by identifying the kitchen location to the living room (attached or detached), the type of cooking fuel used (Clean fuel -LPG/Biogas/Natural gas/Electric stove or Biomass -coal/lignite, charcoal, wood, straw/shrub/grass, crops, and animal dung), smoking behavior of household members (yes or no), and uses of mosquito coil (yes or no). Smoking frequency was categorized and coded as no smoking-0, smoking one to five times-1, six to ten times-2, and eleven times and more-3.

### Statistical analysis

The questionnaires were checked for completeness of responses before entering the data into the software. IBM SPSS statistics-22 software was used to analyze the data. The descriptive statistics were expressed as percentages and frequencies. Due to the nature of the outcome variable, the logistic regression model was deployed. A *p*-value < 0.05 was considered significant and the confidence interval for the Odd Ratio (OR) was set to 95% (95% CI). To identify the independent effect of the explanatory variables on ARI bivariate logistic regression was performed.. A total of three main models were specified to see the adjusted effect of the independent variables on ARI. Each independent variable was tested for multicollinearity prior to multivariable analysis (Table 7). The mean VIF for adjusted model-1 was 1.86 (Min VIF-1.65 Max VIF-2.24), for adjusted model-2 was 1.81(Min VIF-1.06 Max VIF-2.69) and for adjusted model-3 was 1.78 (Min VIF-1.04 Max VIF-2.85). Previous studies reported that a mean VIF of less than 5.0 is acceptable^25,26^. All the adjusted regression models were selected based on the Hosmer and Lemeshow goodness of fit test (Model-1: Chi-square-2.92, df-4, *p*-value- 0.571; Model-2: Chi-square-14.0, df-8, *p*-value- 0.082 and Model -3: Chi-square-9.19, df-8, *p*-value- 0.326). The Hosmer and Lemeshow goodness of fit test results show small chi-squared values with larger *p*-values (*p*-value > 0.05) indicating that the model’s estimate fits the data at an acceptable level^27,28^.

### Ethical approval

We conducted the study according to the guidelines laid down in the Declaration of Helsinki and all procedures involving human subjects were approved by the Institutional Ethical Approval Committee (IEAC) at Primeasia University in Dhaka, Bangladesh. (PAU/IEAC/23/117). All methods were carried out in accordance with the relevant guidelines and regulations. Written informed consent was collected from the mother of the child before data collection.

## Results

### Association between housing infrastructure and demographic variables

An investigation was conducted to examine the association between housing infrastructure and demographic characteristics and the results were given in supplementary materials. The results showed that households constructed with natural floor, wall, and roof materials were common in rural areas. It was observed that household crowding (PPB > 3.0) was more prevalent in households with natural roof types. Less monthly income, in-house smoking, use of biomass as cooking fuel, unimproved toilet facilities, use of shared latrines, and unimproved sources of water are more prevalent in households with natural floor, wall, and roof types.

### Nutritional and birth history of cases and control

About one-third of the cases were born as preterm babies (born at ≤ 37 weeks) and 44.3% of cases were born through C-section. Ninety-two (41.63%) cases were breastfed after one or two days, compared to 45(20.36%) controls; *p* < 0.001 (Table 4).

**Table 4:** Socio-demographic and children’s factors of children under 5 years of age with associations to ARI.

Variables	Category	Case (%)	Control (%)	COR (95% CI)	*p*-value
Gender					
	Male	102 (46.1)	116 (52.5)	1	
	Female	119 (53.9)	105 (47.5)	1.29 (0.89–1.87)	0.183
Mother profession					
	Housewife	118 (81.9)	147 (66.5)	1	
	Day labour	8 (3.6)	19 (8.6)	0.34 (0.16–0.80)	0.014*
	Job	26 (11.8)	37 (16.7)	0.57 (0.33–0.96)	0.044*
	Business	6 (2.7)	18 (8.2)	0.27 (0.10–0.69)	0.007*
Father profession					
	Unemployed	27 (12.2)	59 (26.7)	1	
	Day labour	103 (46.6)	61 (27.6)	3.69 (2.12–6.43)	0.000**
	Job holder	58 (26.2)	61 (27.6)	2.08 (1.63–3.71)	0.013*
	Business	33 (14.9)		1.80 (0.94–3.45)	0.075
Household monthly income (BDT)					
	≤ 10,000	53 (23.98)	64 (28.96)	1	
	10,000–25,000	102 (46.15)	82 (37.1)	1.50 (0.94–2.39)	0.087
	> 25,000	66 (29.86)	75 (33.9)	1.06 (0.65–1.74)	0.809
Gestational age					
	Full-term	152 (68.8)	167 (75.6)	1	
	Preterm (born at ≤ 37 weeks)	69 (31.2)	54 (24.4)	1.40 (0.92–2.013)	0.112
Mode of delivery					
	Normal delivery	123(55.7)	134 (60.6)	1	
	C-section	98 (44.3)	87 (39.4)	1.22 (0.84–1.79)	0.289
Initiation of Breastmilk at born					
	Within one hour	129 (58.37)	176 (79.64)	1	
	After one or two days	92 (41.63)	45 (20.36)	2.79 (1.83–4.26)	0.000**
Source of water					
	Unimproved	94 (42.5)	69 (31.2)	1	
	Improved	127 (57.5)	152 (68.8)	0.61 (0.42–0.91)	0.014*
In-house crowding					
	≤ 3 PPB	159 (71.95)	152 (68.8)	1	
	> 3 PPB	62 (28.05)	69 (31.2)	0.86 (0.57–1.29)	0.466

*COR* Crude odd ratio, *CI* Confidence interval, 1- Reference category.

*p value < 0.05.

**p value < 0.01.

### Environmental and housing factors of cases and control

The results show that a significant number of cases belonged in households made with natural floor, wall, and roofing materials (Table 5). Using biomass as cooking fuel (47.5%), in-house smoking by family members (38.0%), Uses of mosquito coils (54.3%) were common in households with cases compared to controls (Table 6).

**Table 5:** Housing quality and composite indicators with association to ARI.

Variables	Category	Case (%)	Control (%)	COR (95% CI)	*p*-value
Household floor type					
	Finished	124 (56.1)	151 (68.3)	1	
	Natural	97 (43.9)	70 (31.7)	1.69 (1.14–2.49)	0.008*
Household wall type					
Finished	120 (54.3)	132 (59.7)	1		
Natural	101 (45.7)	89 (40.3)	1.25 (0.86–1.82)	0.249	
Household Roof type					
	Finished	91 (41.2)	117 (52.9)	1	
	Natural	130 (58.8)	104 (47.1)	1.61 (1.10–2.34)	0.013*
Composite indicator					
Household floor and wall type					
	Finished floor + finished wall	106 (47.96)	118 (53.39)	1	
	Natural floor + natural wall	83 (37.56)	56 (25.34)	1.65 (1.07–2.53)	0.022*
	Finished floor + natural wall	18 (8.14)	33 (14.93)	0.61 (0.32–1.14)	0.121
	Natural floor + finished wall	14 (6.33)	14 (6.33)	1.11 (0.51–2.44)	0.789
Household wall and roof type					
	Finished wall + finished roof	85 (38.46)	102 (46.15)	1	
	Natural wall + natural roof	95 (42.99)	74 (33.48)	1.54 (1.01–2.34)	0.043*
	Finished wall + natural roof	35 (15.84)	30 (13.57)	1.4 (0.79–2.47)	0.244
	Natural wall^#^ + finished roof	6 (2.71)	15 (6.79)	0.48 (0.19–1.29)	0.146
Household floor and roof type					
	Finished floor + finished roof	75 (33.94)	101 (45.7)	1	
	Natural floor + natural roof	81 (36.65)	54 (24.43)	2.02 (1.28–3.19)	0.003*
	Natural floor + Finished roof	16 (7.24)	16 (7.24)	1.37 (0.63–2.86)	0.439
	Finished floor + natural roof	49 (22.17)	50 (52.62)	1.32 (0.80–2.16)	0.271

*COR* Crude odd ratio, *CI* Confidence interval, 1- Reference Category.

*p -value < 0.05.

**Table 6:** Indoor air pollution in association with ARI.

Variables	Category	Case (%)	Control (%)	COR (95% CI)	*p*-value
Attached kitchen					
	No	72 (32.6)	70 (31.7)	1	
	Yes	149 (67.4)	151 (68.3)	0.96 (0.64–1.43)	0.839
Cooking fuel type					
	Clean fuel	116 (52.5)	139 (62.9)	1	
	Biomass	105 (47.5)	82 (37.1)	1.53 (1.04–2.24)	0.027*
In-house smoking habit by family members					
	No	137 (62.0)	180 (81.5)	1	
	Yes	84 (38.0)	41 (18.5)	2.69 (1.74–4.16)	0.000**
Smoking frequency					
	No smoking	144 (65.2)	173 (78.3)	1	
	1–5 times per day	46 (20.8)	32 (14.5)	1.72 (1.04–2.85)	0.033*
	6–10 times per day	18 (8.1)	13 (5.9)	1.66 (0.79–3.51)	0.182
	11 times and more	13 (5.9)	3 (1.3)	5.21 (1.45–8.63)	0.011*
Use of mosquito coil					
	No	101 (45.7)	119 (53.9)	1	
	Yes	120 (54.3)	102 (46.1)	1.39 (0.95–2.01)	0.087

*COR* Crude odd ratio, *CI* Confidence interval, 1- Reference category.

*p -value < 0.05.

**p -value < 0.01.

### Unadjusted analysis: socio-demographic and children’s factors associated with ARI

The descriptive and unadjusted odds ratio (COR) of socio-demographic and children’s factors are given in Table 4. In this study, children whose mothers were designated as being housewives were more likely to be affected by ARI compared to those whose mothers were designated as being day laborers (COR: 0.34, 95% CI 0.16–0.80), Job holders (COR: 0.57, 95% CI 0.33–0.96) and Business (COR: 0.27, 95% CI 0.10–0.69). On the other hand, children whose fathers were day laborers and job holders had 3.69 times and 2.08 times higher odds respectively to be cases than those whose fathers were designated as unemployed. Initiation of breastmilk after one or two days of childbirth (COR-2.79, 95% CI 1.83–4.26) was significantly associated with ARI cases. Mode of delivery and gestational age did not impact childhood ARI in the unadjusted analysis. Improved water source (COR-0.61, 95% CI 0.42–0.91) is shown to be highly protective against ARI status.

### Unadjusted analysis: housing infrastructure and indoor air pollution associated with childhood ARI

Table 5 shows the unadjusted result of the association of housing infrastructure with ARI symptoms among children under the age of five. The results show that the odds of having ARI symptoms are higher by 69% (COR: 1.69, 95% CI 1.14–2.49) for those who live in a house with natural floor types (vs finished floor type). Household with natural roof type has a significant impact on the ARI status (COR: 1.61, 95% CI 1.10–2.34). However, households with natural walls did not impact on ARI status of children under the age of five. The composite indicator analysis of housing quality show that, natural floor + natural wall (COR: 1.65, 95% CI 1.07–2.53), natural wall + natural roof (COR:1.54, 95% CI 1.01–2.34), and natural floor + natural roof (COR: 2.02, 95% CI 1.28–3.19) were significantly associated with childhood ARI status. The odds of having ARI symptoms are higher by 53% (COR: 1.53, 95% CI 1.04–2.24) for children in households in which biomass is used as cooking fuel. In-house, smoking habit is significantly associated (COR: 2.69, 95% CI 1.74–4.16) with the development of ARI (Table 6).

### Adjusted analysis: multivariable logistic regression

Adjusted odds ratios (AOR) were estimated using three multivariable logistic regression models adjusted for potential confounders (Table 7). The results in Model-1 show that households with the natural floor (AOR: 1.84, 95% CI 1.10–3.07) and roof type (AOR: 1.69, 95% CI 1.04–2.75) were significantly associated with ARI symptoms. However, model 2 suggests that for those who live in houses with natural walls (vs finished walls), the odds of children having ARI symptoms are lower by 47% (AOR: 0.53, 95% CI 0.29–0.98). The results related to the natural floor type and natural roof type of model-2 and model-3 are aligned with model-1 (vs finished product). The results of model-3 also show that cooking stations attached to the living room are associated with higher odds of having ARI symptoms (AOR:1.98, 95% CI 1.08–3.61) than detached kitchens. In-house smoking habits by family members are shown highly associated with ARI symptoms in both model 2 and model 3.

**Table 7:** Multivariable logistic regression analysis assessing the impact of housing infrastructure on ARI of under-five children.

Variables	Model-1	VIF	Model-2	VIF	Model-3	VIF
	AOR (95% CI)		AOR (95% CI)		AOR (95% CI)	
*Household floor type*						
Finished	1	1.68	1	2.69	1	2.85
Natural	1.84 (1.10–3.06)*		2.25 (1.15–4.42)*		2.66 (1.27–5.57)*	
*Household wall type*						
Finished	1	2.24	1	2.27	1	2.31
Natural	0.61 (0.34–1.09)		0.53 (0.29–0.98)*		0.55 (0.28–1.04)	
*Household roof type*						
Finished	1	1.65	1	1.76	1	1.87
Natural	1.69 (1.04–2.75)*		1.96 (1.16–3.32)*		1.78 (1.01–3.11)*	
*Attached kitchen*						
No			1	1.57	1	1.69
Yes			1.64(0.96–2.81)		1.98 (1.08–3.61)*	
*Cooking fuel type*						
Clean Fuel			1	2.23	1	2.53
Biomass			1.19 (0.66–2.17)		1.11 (0.57–2.21)	
*In-house smoking habit by family members*						
No			1	1.06	1	1.06
Yes			2.53 (1.61–3.97)*		2.72 (1.68–4.41)*	
*Use of mosquito coil*						
No			1	1.09	1	1.11
Yes			1.34 (0.89–2.02)		1.30 (0.84–2.02)	
*Mother profession*						
Housewife					1	1.40
Day labour					0.44 (0.14–1.39)	
Job					1.06 (0.47–2.39)	
Business					0.53 (0.16–1.75)	
*Father profession*						
Unemployed					1	1.51
Day labour					2.32 (0.94–5.74)	
Job holder					1.69 (0.69–4.10)	
Business					1.47 (0.55–3.94)	
I*nitiation of breastmilk at born*						
Within one hour					1	1.04
After one or two days					2.65 (1.67–4.19)*	
*Source of water*						
Unimproved					1	2.19
Improved					0.88 (0.46–1.71)	

*AOR* Adjusted odd ratio, *CI* Confidence interval, 1- Reference Category, *VIF* Variance inflation factor.

*p -value < 0.05.

Hosmer and Lemeshow goodness of fit test: Model-1: Chi-square-2.92, df-4, *p*-value- 0.571; Model-2: Chi-square-14.0, df-8, *p*-value- 0.082; Model -3: Chi-square-9.19, df-8, *p*-value- 0.326.

## Discussion

This retrospective study examined the association between housing infrastructure and ARI status among children under five in a tertiary-level hospital in Bangladesh. It was found that children who live in houses with natural flooring have 69% (COR: 1.69, 95% CI 1.14–2.49) higher risk of having ARI symptoms. The underlying cause is that children under the age of five who have a dirt floor are mostly associated with poor health conditions^9,29^. Studies showed that children who live in households made with finished floor products like cement/brick are significantly less likely to experience symptoms of respiratory infection^9,16,29^. The results of this study are aligned with multiple studies carried out in different countries. In Pakistan^11^, Lao PDR^30^, India^15,31^, and Nigeria^32^ the studies found a significant correlation between childhood ARI and homes with natural flooring.

Wall material was not found to be a significant predictor of childhood ARI in the unadjusted model. After being adjusted for indoor air pollution determinants, the natural wall was seen as protective against the occurrence of ARI symptoms (Model-2: AOR-0.53, 95% CI 0.29–0.98). The outcome aligns with the study conducted in Pakistan^11^. The natural wall and fume generated by indoor smoking or cooking activities may be connected because natural walls have more openings than cemented ones which could reduce indoor fume load. More investigation is required to confirm the association between natural walls and ARI status among under-five children.

The current study also suggests that children who live in households with natural roofs have higher odds of having ARI symptoms. As in Bangladesh ‘tin’ makes up the majority of residential roofs, the result of the study could describe ‘tin’ as a significant risk factor for ARIs. Knudsen et al*.*^33^ stated that metal-roofed houses in Asia and Africa are often hotter than thatched ones and create an uncomfortable environment for living. Another study conducted in Nigeria reported that a significant number of kids with ARIs live in houses with wet roofs. The wet roofs could be a result of a leaking roof or a high environmental moisture content^16^. A USA-based international metal company named Sheffield Metals International states from their past experiences that metal roofs including tin could be leaked and vulnerable to corrosion which could allow rainwater, snow, or cold air into the house^34^. These may affect childhood ARI symptoms. More longitudinal studies are required to confirm the relationship between metal roofing and ARI status in under five children.

In terms of passive smoking, the home where the children are more vulnerable^35,36^. Exposure to passive smoke has been reported to be associated with respiratory infections such as pneumonia, wheezing, and coughing^37–39^. The results of the current study show children who were exposed to passive smoking had an increased risk of developing ARI symptoms (vs not exposed). It was found that households with indoor kitchens pose a greater risk of developing ARI symptoms among under-five children compared to households with outdoor kitchens (Model-3, AOR:1.98, 95% CI 1.08–3.61). Furthermore, children who reside in homes using biomass as cooking fuel have a 53% higher risk of developing ARI symptoms. The findings are similar to the studies conducted in Afghanistan^40^ and Ethiopia^6^. This is most likely caused by cooking fuel pollution, indoor cooking activities, and inadequate ventilation, which is linked to the children's respiratory infection^41^. Two factors make young children especially susceptible to fuel pollution: Initially, they typically remain with their mothers while cooking, which causes them to breathe large loads of particulate emissions. A systematic review shows that children's exposure to particle emissions is similar to their mothers^42^. Second, the still-developing bodies of young children make them more vulnerable to ARI than adults, which contributes to the high fatality rate in this age group^43^.

Early initiation of breastfeeding has been shown to be protective against the development of acute respiratory infection symptoms in children under the age of five. This is because colostrum found in milk during the first few hours creates a strong foundation of life-long immunity and improves a child's cognitive function^44–46^. An observational study in Bangladesh has found that the risk of developing severe illness among children increases with an increased delay in breastfeeding initiation^47^. Regarding the mother’s profession, the study showed that children of housewives’ mothers had a higher risk of developing acute respiratory symptoms. This result is consistence with other studies conducted in Ethiopia and Nigeria^6,48,49^. Housewife mothers spend most of their time in the kitchen, preparing meals, and cleaning houses, all of which put small children at risk of exposure to dust and smoke. Another study from Ethiopia stated that professional mothers are more protective against occurring ARI among their children ^50^. Professional mothers are more likely to be educated, which could encourage good home hygiene and shield kids from ARIs.

### Strengths and weaknesses of the study

The findings of the study make an addition to the growing body of literature on household construction materials and their impact on childhood ARI. The strength of the study lies in the use of the MICS category of housing construction materials which is globally recognized. Another strength of this study was that it evaluated important determinant factors including socio-economic status, maternal and child nutrition, and environmental factors contributing to ARI in this case–the control study design. However, there were some limitations to this study. As questionnaires were used to collect the data, recall bias may have impacted the accuracy of the data. The study was conducted among patients who visited the hospital and thus didn’t capture the hidden epidemic that includes caregivers-child pairs who could not use the hospital facilities. Furthermore, the level of indoor air pollution and the ventilation facilities of both natural and finished construction materials households were not measured.

## Conclusion and policy implication

ARIs continue to contribute to a high disease burden among under-five children in Bangladesh.

Poor housing construction materials associated with other household factors significantly influenced the ARI prevalence, thus making it a double burden for families of low economic status. The evidence in this study is an important factor that suggests policy efforts should take improving housing construction materials into stronger consideration. The recommended policy of this study is to replace the poor-quality housing materials. The government could follow Mexico’s anti-poverty cash transfer program (Piso Firme) which replaces dirt floors with cemented ones in low-income families, which has a positive impact on child health^29^. However, behavioral change communication programs encouraging the use of quality house construction materials with health education programs are the interventions the government could undertake to reduce the ARI prevalence in Bangladesh.

### Supplementary Information

Supplementary Information.

## Data Availability

The datasets of the current study will be shared upon request to the corresponding author.
