# Supplementary material for: Effect of housing construction material on childhood acute respiratory infection: a hospital based case control study in Bangladesh
Source: Sci Rep. 2024 Apr 8;14:8163. doi: 10.1038/s41598-024-57820-6 (PMC11001851; doi:10.1038/s41598-024-57820-6)
Supplement: Supplementary file 1 — Supplementary Information. [file 41598_2024_57820_MOESM1_ESM.pdf]

Bivariate Analysis

Tables	Page. No.
Bivariate table 1: Housing infrastructure and in house crowding (PPB-Person Per Bedroom)	1
Bivariate table 2: Housing infrastructure and in house smoking habit by the family members	1
Bivariate table 3: Housing infrastructure and initiation of breastfeeding	2
Bivariate table 4: Housing infrastructure and mode of delivery	2
Bivariate table 5: Housing infrastructure and nutritional status of the children	2
Bivariate table 6: Housing infrastructure and Vaccination (Age specific)	2
Bivariate table 7: Housing infrastructure and gestational age	3
Bivariate table 8: Housing infrastructure and household monthly income	3
Bivariate table 9: Housing infrastructure and location of the residence	3
Bivariate table 10: Housing infrastructure and attachment of kitchen	4
Bivariate table 11: Housing infrastructure and Type of cooking fuel	4
Bivariate table 12: Housing infrastructure and household toilet facilities	4
Bivariate table 13: Housing infrastructure and household condition of shared latrine	4
Bivariate table 14: Housing infrastructure and household source of water	5

Bivariate table 1: Housing infrastructure and in house crowding (PPB-Person Per Bedroom)

	In-house crowding		Total
Floor type	≤ 3 PPB	>3 PPB	
Finished	205(65.92%)	70(53.44%)	275(62.22%)
Natural	106(34.08%)	61(46.56%)	167(37.78%)
Wall Type			
Finished	195(62.7%)	57(43.51%)	252(57.01%)
Natural	116(37.3%)	74(56.49%)	190(42.99%)
Roof Type			
Finished	157(50.48%)	51(38.93%)	208(47.06%)
Natural	154(49.52%)	80(61.07%)	234(52.94%)

Bivariate table 2: Housing infrastructure and in house smoking habit by the family members

	in house smoking habit by the family members		Total
Floor type	No	Yes	
Finished	199(62.78%)	76(60.80%)	275(62.22%)
Natural	118(37.22%)	49(39.20%)	167(37.78%)
Wall Type			
Finished	185(58.36%)	67(53.60%)	252(57.01%)
Natural	132(41.64%)	58(46.40%)	190(42.99%)
Roof Type			
Finished	153(48.26%)	55(44.0%)	208(47.06%)
Natural	164(51.74%)	70(56.0%)	234(52.94%)

Bivariate table 3: Housing infrastructure and initiation of breastfeeding

	initiation of breastfeeding		Total
Floor type	Within one hour	After one or two days	
Finished	193(63.28%)	82(59.85%)	275(62.22%)
Natural	112(36.72%)	55(40.15%)	167(37.78%)
Wall Type			
Finished	174(57.05%)	78(56.93%)	252(57.01%)
Natural	131(42.95%)	59(43.07%)	190(42.99%)
Roof Type			
Finished	151(49.51%)	57(41.61%)	208(47.06%)
Natural	154(50.49%)	80(58.39%)	234(52.94%)

Bivariate table 4: Housing infrastructure and mode of delivery

	Mode of delivery		Total
Floor type	Normal Delivery	C-section	
Finished	137(53.31%)	138(74.59%)	275(62.22%)
Natural	120(46.69%)	47(25.41%)	167(37.78%)
Wall Type			
Finished	112(43.58%)	140(75.68%)	252(57.01%)
Natural	145(56.42%)	45(24.32%)	190(42.99%)
Roof Type			
Finished	93(36.19%)	115(62.16%)	208(47.06%)
Natural	164(63.81%)	70(37.84%)	234(52.94%)

Bivariate table 5: Housing infrastructure and nutritional status of the children

	MUAC			Total
Floor type	Healthy	Moderate acute malnourished	Severe acute malnourished	
Finished	223(64.45%)	40(50.0%)	12(75.0%)	275(62.22%)
Natural	123(35.55%)	40(50.0%)	4(25.0%)	167(37.78%)
Wall Type				
Finished	201(58.09%)	41(51.25%)	10(62.50%)	252(57.01%)
Natural	145(41.91%)	39(48.75%)	6(37.50%)	190(42.99%)
Roof Type				
Finished	164(47.40%)	33(41.25%)	11(68.75%)	208(47.06%)
Natural	182(52.60%)	47(58.75%)	5(31.25%)	234(52.94%)

Bivariate table 6: Housing infrastructure and Vaccination (Age specific)

	Vaccination		Total
Floor type	Not completed	Completed	
Finished	22(57.89%)	253(62.62%)	275(62.22%)
Natural	16(42.11%)	151(37.38%)	167(37.78%)
Wall Type			
Finished	20(52.63%)	232(57.43%)	252(57.01%)

Natural	18(47.37%)	172(42.57%)	190(42.99%)
Roof Type			
Finished	13(34.21%)	195(48.27%)	208(47.06%)
Natural	25(65.79%)	209(51.73%)	234(52.94%)

Bivariate table 7: Housing infrastructure and gestational age

	Born before 37 weeks		Total
Floor type	No	Yes	
Finished	215(67.40%)	60(48.78%)	275(62.22%)
Natural	104(32.60%)	63(51.22%)	167(37.78%)
Wall Type			
Finished	191(59.87%)	61(49.59%)	252(57.01%)
Natural	128(40.13%)	62(50.41%)	190(42.99%)
Roof Type			
Finished	158(49.53%)	50(40.65%)	208(47.06%)
Natural	161(50.47%)	73(59.35%)	234(52.94%)

Bivariate table 8: Housing infrastructure and household monthly income

	Household monthly income			Total
Floor type	≤10,000 BDT	10,001-25000 BDT	>25,000 BDT	
Finished	43(36.75%)	102(55.43%)	130(92.20%)	275(62.22%)
Natural	74(63.25)	82(44.57%)	11(7.80%)	167(37.78%)
Wall Type				
Finished	25(21.37%)	97(52.72%)	130(92.20%)	252(57.01%)
Natural	92(78.63%)	87(47.28%)	11(7.80%)	190(42.99%)
Roof Type				
Finished	15(12.82%)	82(44.57%)	111(78.72%)	208(47.06%)
Natural	102(87.18%)	102(55.43%)	30(21.28%)	234(52.94%)

Bivariate table 9: Housing infrastructure and location of the residence

	location of the residence		Total
Floor type	Rural	Urban	
Finished	103(39.92%)	172(93.48%)	275(62.22%)
Natural	155(60.08%)	12(6.52%)	167(37.78%)
Wall Type			
Finished	84(32.56%)	168(91.30%)	252(57.01%)
Natural	174(67.44%)	16(8.70%)	190(42.99%)
Roof Type			
Finished	70(27.13%)	138(75.0%)	208(47.06%)
Natural	188(72.87%)	46(25.0%)	234(52.94%)

Bivariate table 10: Housing infrastructure and attachment of kitchen

	Attached kitchen		Total
Floor type	No	Yes	
Finished	39(27.46%)	236(78.67%)	275(62.22%)
Natural	103(72.54%)	64(21.33%)	167(37.78%)
Wall Type			
Finished	48(33.80%)	204(68.0%)	252(57.01%)
Natural	94(66.20%)	96(32.0%)	190(42.99%)
Roof Type			
Finished	27(19.01%)	181(60.33%)	208(47.06%)
Natural	115(80.99%)	119(39.67%)	234(52.94%)

Bivariate table 11: Housing infrastructure and Type of cooking fuel

	Types of fuel used for cooking		Total
Floor type	Clean fuel/LPG	Biomass/Wood	
Finished	233(91.37%)	42(22.46%)	275(62.22%)
Natural	22(8.63%)	145(77.54%)	167(37.78%)
Wall Type			
Finished	195(76.47%)	57(30.48%)	252(57.01%)
Natural	60(23.53%)	130(69.52%)	190(42.99%)
Roof Type			
Finished	160(62.75%)	48(25.67%)	208(47.06%)
Natural	95(37.25%)	139(74.33%)	234(52.94%)

Bivariate table 12: Housing infrastructure and household toilet facilities

	Toilet facilities		Total
Floor type	Improved	Unimproved	
Finished	264(67.18%)	11(22.45%)	275(62.22%)
Natural	129(32.82%)	38(77.55%)	167(37.78%)
Wall Type			
Finished	242(61.58%)	10(20.41%)	252(57.01%)
Natural	151(38.42%)	39(79.59%)	190(42.99%)
Roof Type			
Finished	196(49.87%)	12(24.49%)	208(47.06%)
Natural	197(50.13%)	37(75.51%)	234(52.94%)

Bivariate table 13: Housing infrastructure and household condition of shared latrine

	Shared latrine		Total
Floor type	No	Yes	
Finished	178(62.02%)	97(62.58%)	275(62.22%)
Natural	109(37.98%)	58(37.42%)	167(37.78%)
Wall Type			
Finished	187(65.16%)	65(41.94%)	252(57.01%)
Natural	100(34.84%)	90(58.06%)	190(42.99%)

Roof Type			
Finished	171(59.58%)	37(23.87%)	208(47.06%)
Natural	116(40.42%)	118(76.13%)	234(52.94%)

Bivariate table 14: Housing infrastructure and household source of water

	Household source of water		Total
Floor type	Unimproved	Improved	
Finished	39(23.93%)	236(84.59%)	275(62.22%)
Natural	124(76.07%)	43(15.41%)	167(37.78%)
Wall Type			
Finished	48(29.45%)	204(73.12%)	252(57.01%)
Natural	115(70.55%)	75(26.88%)	190(42.99%)
Roof Type			
Finished	29(17.79%)	179(64.16%)	208(47.06%)
Natural	134(82.21%)	100(35.84%)	234(52.94%)
